# Evaluation of zirconia and titanium abutments on peri-implant tissues: A comparative study

**DOI:** 10.6026/9732063002001809

**Published:** 2024-12-31

**Authors:** Swatantra Kumar, Alakesh Singha, Jatin Dhingra, Gaurav Verma, Sachin Dev Singh, Amit Kumar

**Affiliations:** 1 Department of Prosthodontics Crown and Bridge and Implantology, Awadh Dental College and Hospital, Jamshedpur, Jharkhand, India; 2 Department of Conservative Dentistry & Endodontics, Private Practitioner at Alaka's Odontocare Dental Clinic & Endodontic Centre, Agartala , Tripura, India; 3 Department of Oral and Maxillofacial Surgery, Himachal Institute of Dental Sciences, Paonta Sahib, Himachal Pradesh, India; 4 Department of Oral and Maxillofacial Surgery, Kothiwal Dental College and Research Centre, Moradabad, Uttar Pradesh, India; 5 Department of Oral and Maxillofacial Surgery, Teerthanker Mahaveer Dental College and Research Centre, Moradabad, Uttar Pradesh, India; 6 Department of Prosthodontics Crown and Bridge and Implantology, Seema Dental College and Hospital, Rishikesh, Uttarakhand, India

**Keywords:** Zirconia abutments, titanium abutments, peri-implant tissues, dental implants, marginal bone loss

## Abstract

The comparative effect of zirconia and titanium abutments on peri-implant hard and soft tissues is of interest to dentists. Hence, a total of 40 patients with single-tooth implants in the posterior region were selected and divided into two groups: zirconia abutments (Group A, n=20) and titanium abutments (Group B, n=20). Clinical parameters, including probing depth (PD), bleeding on probing (BOP) and peri-implant marginal bone level (MBL), were evaluated at baseline, 3 months and 6 months post-implant placement. Radiographic analysis was used to measure changes in marginal bone levels. Zirconia abutments showed a more favourable outcome on peri-implant soft tissue health and marginal bone preservation than titanium abutments. Zirconia abutments further show reduced inflammation and bone resorption around dental implants.

## Background:

Dental implants are widely recognized as a reliable solution for the replacement of missing teeth, with high success rates and long-term clinical stability [1]. However, the health and stability of peri-implant tissues are critical factors in ensuring the longevity and functionality of the implant [2]. The choice of abutment material has been shown to significantly impact the biological response of peri-implant tissues, influencing factors such as soft tissue integration and marginal bone levels [3]. Titanium has long been considered the gold standard for implant abutments due to its excellent biocompatibility and mechanical properties [4]. It has demonstrated favourable outcomes in terms of osseointegration and long-term success. However, concerns have been raised regarding its esthetic limitations, particularly in the anterior region, where the greyish hue of titanium can compromise the overall appearance [5]. Moreover, some studies have suggested that titanium abutments may induce a greater inflammatory response in the surrounding soft tissues compared to alternative materials [6]. Zirconia, a highly-strength ceramic material and excellent bio-compatibility, has been introduced as an alternative to titanium abutments. Its tooth-like colour and superior esthetic properties make it particularly appealing in cases where esthetic a primary concern [7]. In addition to its esthetic advantages, zirconia has been reported to exhibit lower plaque accumulation and bacterial adhesion, which may contribute to healthier peri-implant soft tissues [8]. Some studies have suggested that zirconia abutments may result in better soft tissue response and less marginal bone loss compared to titanium abutments [9, 10]. Despite the growing body of evidence, more comprehensive studies are needed to compare the effects of zirconia and titanium abutments on peri-implant tissues. This study evaluates and compares the impact of zirconia and titanium abutments on peri-implant hard and soft tissues over six months.

## Materials and Methods:

## Study design and patient selection:

This prospective clinical study included a total of 40 patients requiring single-tooth implant restorations in the posterior region. Patients were selected based on the following inclusion criteria:

[1] Age between 20 and 60 years

[2] Non-smokers or light smokers (less than 10 cigarettes per day)

[3] Adequate bone volume for implant placement without the need for bone grafting

[4] Good oral hygiene, with a full-mouth plaque score (FMPS) and full-mouth bleeding score (FMBS) of less than 20%

[5] Absence of systemic diseases that could affect implant healing

Exclusion criteria included patients with uncontrolled systemic conditions, heavy smokers, that requiring bone augmentation and patients with a history of periodontal disease. The institutional ethics committee approved the study protocol and written informed consent was obtained from all participants.

## Clinical and radiographic evaluation:

Clinical parameters were assessed at baseline (abutment placement), 3 months and 6 months post-abutment placement. The following clinical parameters were recorded:

[1] Probing Depth (PD): Measured at four sites around each implant using a periodontal probe.

[2] Bleeding on Probing (BOP): Recorded as present or absent at each site.

[3] Plaque Index (PI): Assessed at four sites around each implant.

The radiographic evaluation measured changes in peri-implant marginal bone level (MBL). Standardized periapical radiographs were taken at baseline, 3 months and 6 months. Marginal bone levels were measured using image analysis software, with measurements taken from a reference point on the implant (implant-abutment junction) to the most coronal point of bone-to-implant contact on both the mesial and distal aspects.

## Statistical analysis:

Data were analyzed using statistical software. The mean and standard deviation (SD) for each parameter were calculated for both groups. The changes in PD, BOP, PI and MBL between baseline, 3 months and 6 months were compared using the paired t-test for intragroup comparisons and the independent t-test for intergroup comparisons. A p-value of <0.05 was considered statistically significant.

## Results:

## Clinical parameters:

The clinical parameters, including probing depth (PD), bleeding on probing (BOP) and plaque index (PI), were evaluated at baseline, 3 months and 6 months for both groups (Table 1).

## Radiographic analysis:

Marginal bone levels (MBL) were measured at baseline, 3 months and 6 months using standardized periapical radiographs (Table 2).

## Summary of findings:

[1] Probing depth (PD): Both groups showed a reduction in PD over time. However, Group A (zirconia) demonstrated a significantly greater reduction in PD at 6 months compared to Group B (titanium) (p = 0.02).

[2] Bleeding on probing (BOP): There was a statistically significant reduction in BOP in the zirconia group compared to the titanium group at both 3 months (p = 0.04) and 6 months (p = 0.03).

[3] Plaque index (PI): Group A exhibited a lower PI score at 6 months, indicating better soft tissue health around zirconia abutments compared to titanium (p = 0.01).

[4] Marginal bone level (MBL): Group A (zirconia) showed less marginal bone loss compared to Group B (titanium) at 6 months, with a statistically significant difference (p = 0.01).

Zirconia abutments demonstrated a more favourable effect on peri-implant soft tissue health and marginal bone preservation over the 6-month evaluation period.

## Discussion:

The present study aimed to compare the effects of zirconia and titanium abutments on peri-implant hard and soft tissues. The findings indicate that zirconia abutments are associated with better peri-implant tissue health and reduced marginal bone loss compared to titanium abutments. The reduction in probing depth (PD) observed with zirconia abutments aligns with previous research suggesting that zirconia's favourable surface properties may contribute to improved soft tissue integration [1, 2]. Zirconia has a lower surface roughness than titanium, which may reduce bacterial adhesion and plaque accumulation, decreasing the risk of peri-implantitis [3, 4]. In addition, the reduced inflammation around zirconia abutments could explain the significant reduction in bleeding on probing (BOP) observed in this study [5, 6]. Studies have shown that zirconia exhibits a lower inflammatory response than titanium, possibly due to its superior biocompatibility and minimal release of ions into the surrounding tissues [7, 8].The plaque index (PI) scores in the zirconia group were significantly lower than those in the titanium group, supporting the hypothesis that zirconia abutments promote healthier peri-implant soft tissues [9, 10]. The smooth surface and reduced bacterial colonization associated with zirconia abutments may contribute to this outcome [11]. Furthermore, the esthetic advantages of zirconia, such as its tooth-like colour and translucency, may play a role in the enhanced soft tissue response observed in this study [12, 13]. Previous studies have emphasized the importance of abutment material in achieving optimal esthetic outcomes, particularly in the anterior region where soft tissue health is critical for patient satisfaction [14, 15]. Marginal bone levels (MBL) were better preserved in the zirconia group, with significantly less bone loss compared to the titanium group over the 6-month period [6, 7]. This finding is consistent with previous reports showing that zirconia abutments are less likely to induce peri-implant bone resorption [8, 9]. The reasons for this could be multifactorial, including zirconia's lower affinity for plaque, reduced inflammatory response and possibly better soft tissue seal, which may act as a barrier to microbial invasion and subsequent bone loss [10, 11]. On the other hand, titanium abutments have been associated with higher levels of peri-implant bone resorption, potentially due to the release of titanium particles into the peri-implant tissues and the associated inflammatory response [12, 13]. However, it is important to note that while zirconia abutments appear to have advantages over titanium regarding soft tissue health and marginal bone preservation, both materials showed acceptable clinical outcomes in this study [14, 15]. The choice of abutment material should be made based on individual patient needs, esthetic considerations and long-term clinical success [6, 7]. Further research with longer follow-up periods and larger sample sizes is needed to confirm the long-term benefits of zirconia abutments and their impact on the survival of dental implants [8, 9].

## Conclusion:

Data shows that zirconia abutments have a more favourable effect on peri-implant soft tissue health and marginal bone preservation than titanium abutments. Zirconia abutments show a significant reduction in probing depth and bleeding on probing. Moreover, lower plaque accumulation and marginal bone loss is observed over a 6-month period.

## Figures and Tables

**Table 1:** Clinical parameters (Mean ± SD)

**Time Point**	**Group A (Zirconia)**	**Group B (Titanium)**	**p-value**
**Probing Depth (PD) (mm)**			
Baseline	2.5 ± 0.4	2.6 ± 0.5	0.34
3 Months	2.3 ± 0.3	2.5 ± 0.4	0.05
6 Months	2.0 ± 0.2	2.3 ± 0.3	0.02
**Bleeding on Probing (BOP) (%)**			
Baseline	25%	30%	0.6
3 Months	15%	25%	0.04
6 Months	10%	20%	0.03
**Plaque Index (PI) (Score)**			
Baseline	1.5 ± 0.4	1.6 ± 0.3	0.45
3 Months	1.2 ± 0.3	1.4 ± 0.4	0.07
6 Months	1.0 ±0.2	1.3 ± 0.3	0.01

**Table 2:** Marginal bone level (MBL) changes (Mean ± SD in mm)

**Time Point**	**Group A (Zirconia)**	**Group B (Titanium)**	**p-value**
Baseline	0.0 ± 0.0	0.0 ± 0.0	N/A
3 Months	-0.1 ± 0.1	-0.2 ± 0.2	0.08
6 Months	-0.2 ± 0.1	-0.4 ± 0.2	0.01
Total Change (Baseline to 6 Months)	-0.2 ± 0.1	-0.4 ± 0.2	0.01
